# Comparing the results of manual and automated quantitative corneal neuroanalysing modules for beginners

**DOI:** 10.1038/s41598-021-97567-y

**Published:** 2021-09-14

**Authors:** Po-Ying Wu, Jo-Hsuan Wu, Yi-Ting Hsieh, Lin Chih-Chieh Chen, Ting Cheng, Po-Yi Wu, Bing-Jun Hsieh, Wei-Lun Huang, Sheng-Lung Huang, Wei-Li Chen

**Affiliations:** 1 Department of General Medicine, Kaohsiung Medical University Hospital, Kaohsiung Medical University, Kaohsiung, Taiwan; 2 Shiley Eye Institute and Viterbi Family Department of Ophthalmology, University of California, San Diego, USA; 3 Department of Ophthalmology, National Taiwan University Hospital, No. 7, Chung-Shan South Road, Taipei, Taiwan; 4 Graduate Institute of Photonics and Optoelectronics, National Taiwan University, Taipei, Taiwan; 5 Department of Electrical Engineering, National Taiwan University, Taipei, Taiwan; 6 Advanced Ocular Surface and Corneal Nerve Regeneration Center, National Taiwan University Hospital, Taipei, Taiwan; 7 Department of Ophthalmology, College of Medicine, National Taiwan University, Taipei, Taiwan

**Keywords:** Computational biology and bioinformatics, Neuroscience

## Abstract

This study aimed to evaluate the reliability of in vivo confocal microscopic neuroanalysis by beginners using manual and automated modules. Images of sub-basal corneal nerve plexus (SCNP) from 108 images of 18 healthy participants were analyzed by 7 beginner observers using manual (CCMetrics, [CCM]) and automated (ACCMetrics, [ACCM]) module. SCNP parameters analyzed included corneal nerve fiber density (NFD), corneal nerve branch density (NBD), corneal nerve fiber length (NFL), and tortuosity coefficient (TC). The intra-observer repeatability, inter-observer reliability, inter-module agreement, and left–right eye symmetry level of SCNP parameters were examined. All observers showed good intra-observer repeatability using CCM (intraclass correlation coefficient [ICC] > 0.60 for all), except when measuring TC. Two observers demonstrated especially excellent repeatability in analyzing NFD, NBD, and NFL using manual mode, indicating the quality of interpretation may still be observer-dependent. Among all SCNP parameters, NFL had the best inter-observer reliability (Spearman’s rank-sum correlation coefficient [SpCC] and ICC > 0.85 for the 3 original observers) and left–right symmetry level (SpCC and ICC > 0.60). In the additional analysis of inter-observer reliability using results by all 7 observers, only NFL showed good inter-observer reliability (ICC = 0.79). Compared with CCM measurements, values of ACCM measurements were significantly lower, implying a poor inter-module agreement. Our result suggested that performance of quantitative corneal neuroanalysis by beginners maybe acceptable, with NFL being the most reliable parameter, and automated method cannot fully replace manual work.

## Introduction

The cornea is the most densely innervated tissue of the human body^1^. The sub-basal corneal nerve plexus (SCNP), which locates between the Bowman layer and the corneal basal epithelium, is a homogenous anastomotic plexus of corneal nerves. A lot of ocular and systemic diseases, including diabetic neuropathy^2,3^, dry eye syndrome^4–6^, laser refractive surgery^7–9^ and peripheral neuropathies^10–13^, may lead to impairment of corneal nerves. As corneal innervation is important to the physiology and homeostasis of the cornea, damage to the SCNP may cause detrimental effects to our vision.

In vivo confocal microscopy (IVCM) is a non-invasive clinical tool that helps evaluate microscopic corneal structures^14^, and is especially useful in detecting corneal nerve changes. Multiple quantitative outcome measurements can be derived from IVCM to assess changes in the pattern of corneal innervation, including the length, density, and tortuosity of the nerves^15^. Other less commonly examined morphometric parameters include beading, branching, reflectivity, and fiber diameter^16,17^. All these parameters have been used in past studies to evaluate corneal nerve; however, the most reliable method for quantitative neuroanalysis has not yet been concluded^18–21^.

Currently, quantification of SCNP parameters based on IVCM images relies mainly on manual technique. Since manual work is highly labor intensive, a manual module software, CCMetrics (CCM; Manual tracing of nerve fibers, Manchester University, Manchester, UK), was developed to improve the interpreting efficiency. Automated module software, ACCMetrics (ACCM, Automated tracing of nerve fibers, Manchester University, Manchester, UK), was later developed to perform automatic analysis of nerve parameters. Some previous studies have compared the measurements of CCM and ACCM modules, and the results were not considered interchangeble^18,22^. Due to the non-desirable performance of automated software, some studies recruited less experienced observers or used crowdsourcing to perform large-volume image interpretation, as formal training for neuroanalysis is time- and labor-consuming. Such methods have also been applied to grading diabetic retinopathy and showed satisfactory result^23^.

Before a reliable automated neuroanalytic method for corneal nerve is developed, it is important to investigate if less experienced observers can still achieve acceptable results for corneal neuroanalysis through manual work. In this study, we evaluated the reliability of beginner observers in interpreting IVCM images for measuring SCNP parameters using both manual and automated modules. We aimed to evaluate the reliability of both neuroanalytic modules for beginner observers, and selected the most reliable parameters. Our study may provide precious information and help dealing with the big and complicated data from the corneal nerve images.

## Results

Eighteen male subjects (age: 23.0 ± 1.56 years, range 20 to 26 years) were enrolled for IVCM imaging, and a total of 108 images were used in formal evaluation (3 images per eye per subject).

### Intra-observer repeatability

**Figure 1:**
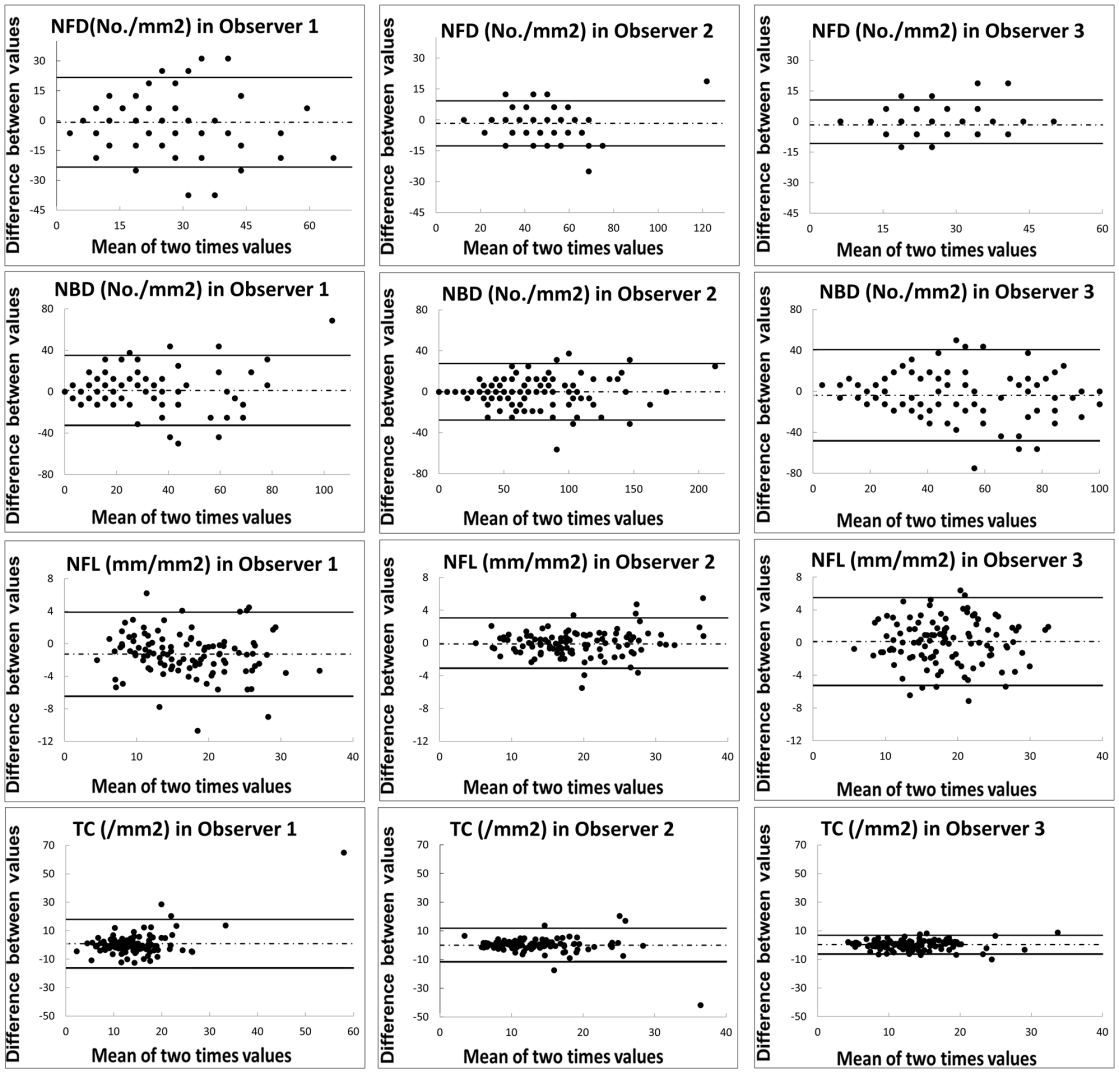
Bland–Altman plots for nerve fiber density (NFD), nerve branch density (NBD), nerve fiber length (NFL), and tortuosity coefficient (TC) to measure intra-observer repeatability in different two evaluations in the original group. The dotted line represents the mean difference, and the solid lines represent the 95% limits of agreement. Observer 2 was found to have the highest repeatability in the original group. In NFL, all three observers reached excellent repeatability.

The CCM measurements and ACCM measurements obtained by the 3 original observers (Original group, Observer 1–3) were summarized in Table 1. The CCM measurements by the 4 additional observers (Additional group, Observer 4–7) were summarized in Supplement Table S1. To evaluate the intra-observer repeatability of individual observer, the intra-class correlation (ICC) was calculated based on data from both sessions of evaluation (Table 2). All 7 observers showed good intra-observer repeatability (ICC > 0.6) when measuring all SCNP parameters, except for corneal tortuosity coefficient (TC). Excellent intra-observer repeatability (ICC > 0.8) was found in 6, 3, 6, 3 out of 7 observers for the analysis of corneal nerve fiber density (NFD), corneal nerve branch density (NBD), corneal nerve fiber length (NFL), and TC respectively, with the best performance in NFL. Two observers (Observer 2 and Observer 5) achieved especially excellent intra-observer repeatability for NFD, NBD, and NFL. Bland–Altman plots, which demonstrated the intra-observer repeatability of each original observer, were shown in Fig. 1.

**Table 1:** The CCMetrics (CCM) values of NFD, NBD and NFL in the first and the second evaluations from the original group and the ACCMetrics (ACCM) values and the values of both eyes from the first visit of the observer 2 and ACCM.

	NFD (no./mm^2^)	NBD (no./mm^2^)	NFL (mm/mm^2^)	TC
**Manual and automated module**				
CCMetrics (CCM), Observer 1				
Evaluation 1	24.02 ± 8.50	25.81 ± 13.28	17.56 ± 5.52	13.62 ± 2.52
Evaluation 2	23.15 ± 8.35	27.03 ± 14.49	16.28 ± 5.08	14.47 ± 3.10
CCMetrics, Observer 2				
Evaluation 1	42.48 ± 9.71	69.21 ± 28.85	18.97 ± 5.72	13.40 ± 3.48
Evaluation 2	40.80 ± 9.75	69.10 ± 28.74	18.96 ± 5.90	13.62 ± 3.19
CCMetrics, Observer 3				
Evaluation 1	24.42 ± 4.80	55.21 ± 23.43	18.27 ± 4.88	12.98 ± 2.96
Evaluation 2	24.36 ± 5.01	51.33 ± 18.90	18.41 ± 4.87	13.28 ± 2.58
ACCMetrics (ACCM)				
Evaluation 1	10.01 ± 5.78	6.37 ± 5.67	7.78 ± 3.21	NA
**Symmetry**				
CCMetrics, Observer 2				
Evaluation 1, OD	41.67 ± 11.94	63.31 ± 28.31	18.99 ± 6.28	13.18 ± 3.41
Evaluation 1, OS	43.29 ± 11.99	75.12 ± 35.90	18.96 ± 5.88	13.62 ± 5.81
ACCMetrics (ACCM)				
Evaluation 1, OD	10.99 ± 6.42	6.25 ± 7.53	7.90 ± 3.48	NA
Evaluation 1, OS	9.03 ± 7.07	6.48 ± 7.07	7.65 ± 3.43	NA

Results are expressed as Mean ± SD.

*NFD* (nerve fiber density) is measured in number of fibers/mm^2^, *NBD* (nerve branch density) is measured in number of branch points on the main fibers/mm^2^, *NFL* (nerve fiber length) is measured in total length of fiber (mm/mm^2^), *TC* (tortuosity coefficient) is measured in main fiber average tortuosity, *OD* (Oculus Dexter) represents right eye, *OS* (Oculus Sinister) represents left eye, *NA* non-applicable.

**Table 2:** The intraclass correlation coefficient for 2 CCMetrics (CCM) measurements separated by 2 weeks in all observers.

	NFD	NBD	NFL	TC
**Intraclass correlation coefficient (95% CI)**				
Observer 1	0.60 (0.47–0.71)	0.72 (0.62–0.80)	0.91* (0.82–0.95)	0.40 (0.23–0.55)
Observer 2	0.93* (0.89–0.95)	0.94* (0.91–0.96)	0.98* (0.96–0.98)	0.56 (0.42–0.68)
Observer 3	0.80* (0.72–0.86)	0.72 (0.62–0.80)	0.89* (0.85–0.93)	0.81* (0.74–0.87)
Observer 4	0.82* (0.75–0.87)	0.76 (0.54–0.87)	0.90* (0.64–0.96)	0.75 (0.63–0.83)
Observer 5	0.83* (0.76–0.88)	0.91* (0.87–0.94)	0.97* (0.95–0.98)	0.84* (0.77–0.87)
Observer 6	0.80* (0.65–0.88)	0.83* (0.74–0.89)	0.77 (0.67–0.83)	0.85* (0.79–0.90)
Observer 7	0.91* (0.87–0.94)	0.70 (0.58–0.78)	0.80* (0.72–0.86)	0.78 (0.70–0.84)

*NFD* (nerve fiber density) is measured in number of fibers/mm^2^, *NBD* (nerve branch density) is measured in number of branch points on the main fibers/mm^2^, *NFL* (nerve fiber length) is measured in total length of fiber (mm/mm^2^), *TC* (tortuosity coefficient) is measured in main fiber average tortuosity, *CI* confidence interval.

*Represents an excellent correlation when values > 0.8.

### Inter-observer reliability

The results of inter-observer reliability of the original group were summarized in Table 3. The inter-observer ICC was 0.20, 0.39, 0.86, and 0.44 for NFD, NBD, NFL, and TC, respectively. In the 4 parameters, NFL had the best inter-observer reliability (ICC = 0.86 when comparing Observer 1, 2, and 3; SpCC = 0.87 and ICC = 0.86 when comparing Observer 1 and 2; SpCC = 0.87 and ICC = 0.87 when comparing Observer 2 and 3, and SpCC = 0.85 and ICC = 0.85 when comparing Observer 1 and 3). Additionally, we calculated the inter-observer reliability using data by all 7 observers, and a similar result was found (overall ICC = 0.27, 0.39, 0.79, 0.54 for NFD, NBD, NFL, and TC, respectively), with NFL being the only parameter showing a good inter-observer reliability.

**Table 3:** The Spearman correlation coefficient (SpCC) and intraclass correlation coefficient (ICC) to assess inter-observer reliability and inter-module agreement between CCMetrics and ACCMetrics and the left–right eye symmetry for the original group.

	NFD		NBD		NFL		TC	
	SpCC	ICC	SpCC	ICC	SpCC	ICC	SpCC	ICC
**Inter-observer reliability**								
Original group		0.20		0.39		0.86*		0.44
Observer 1 vs Observer 2	0.34	0.18	0.55	0.26	0.87*	0.86*	0.49	0.32
Observer 2 vs Observer 3	0.37	0.11	0.55	0.55	0.87*	0.87*	0.71	0.49
Observer 1 vs Observer 3	0.58	0.50	0.63	0.41	0.85*	0.85*	0.52	0.51
**Inter-module agreement**								
ACCM vs Observer 1	0.53	0.23	0.51	0.22	0.86*	0.29	NA	NA
ACCM vs Observer 2	0.21	0.03	0.47	0.06	0.81*	0.23	NA	NA
ACCM vs Observer 3	0.47	0.21	0.54	0.10	0.83*	0.24	NA	NA
**Symmetry of left–right eye**								
Observer 1	0.47	0.45	0.62	0.54	0.72	0.67	0.03	0.07
Observer 2	0.49	0.39	0.73	0.69	0.86*	0.83*	0.38	0.13
Observer 3	0.30	0.29	0.56	0.55	0.67	0.70	0.13	0.18
ACCM	0.52	0.55	0.29	0.28	0.83*	0.83*	NA	NA

*NFD* (nerve fiber density) is measured in number of fibers/mm^2^, *NBD* (nerve branch density) is measured in number of branch points on the main fibers/mm^2^, *NFL* (nerve fiber length) is measured in total length of fiber (mm/mm^2^), *TC* (tortuosity coefficient) is measured in main fiber average tortuosity, *SpCC* Spearman correlation coefficient, *ICC* intraclass correlation coefficient, *ACCM* ACCMetrics module. The CCM data from the first evaluation in all 3 observers were compared with ACCM data, *NA* not applicable.

*Represents an excellent correlation when values > 0.8.

### CCM module vs. ACCM module

**Figure 2:**
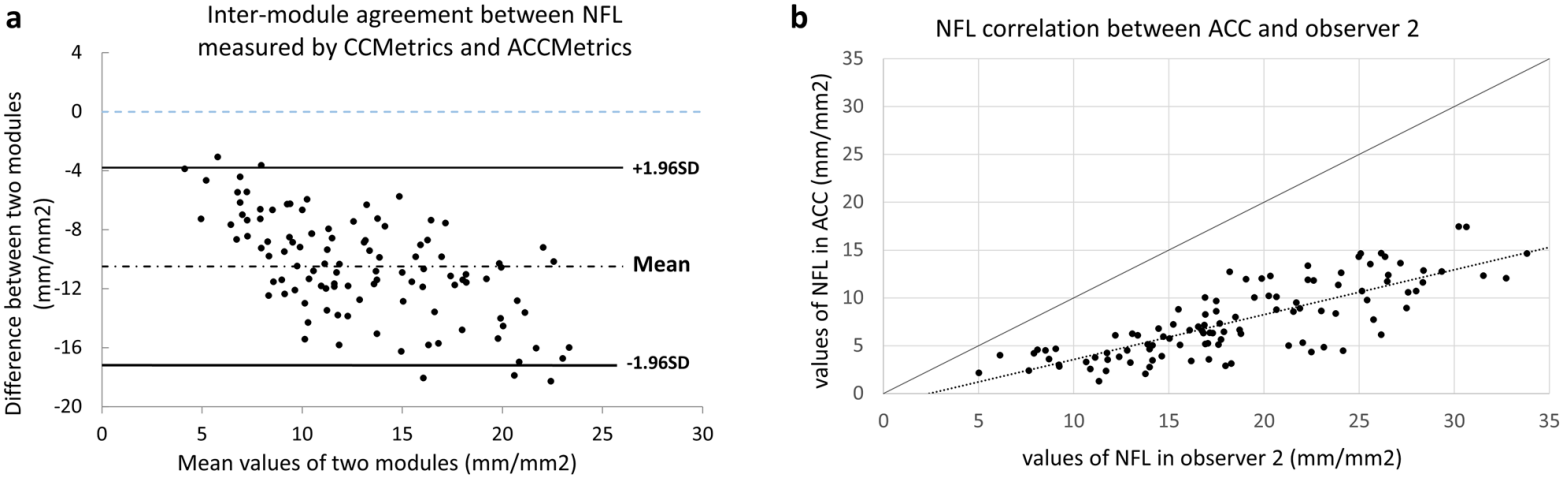
Agreement plot **(a)** and comparison plot **(b)** of nerve fiber length (NFL) between observer 2, the observer with the highest intra-observer repeatability in the original group, and ACCMetrics (ACCM). In **(a)**, the black dotted line represents the mean differences, and the solid lines represent the 95% limits of agreement. The blue dashed line represents no difference between NFL measured by two different modules. In **(b)**, linear regression was depicted in the dotted line and equivalence line was depicted in the solid line. Correlation was excellent between observer 2 and ACCM but there was a significant underestimation using ACCM to calculate NFL comparing with using manual CCMetrics (CCM).

The ACCM-derived values of NFD, NBD and NFL were significantly lower compared to those measured with the CCM module in the first and second evaluation by the original group (*p* all < 0.001 for NFD, NBD and NFL) (Table 1). The results of inter-module agreement of the original group were summarized in Table 3. The ICC of inter-module agreement (ACCM vs either observer 1, 2, or 3) was all < 0.6 for NFD, NBD, and NFL, implying a poor inter-module agreement. Similar results were found when CCM data derived from the additional group was used to compare with ACCM result (ICC of inter-module agreement < 0.6, Supplement Table S2). Agreement plot and linear regression was also performed to visualize the relationship between NFL measurements using CCM by observer 2 and the measurement by ACCM (Fig. 2). The plot demonstrated good correlation between measurement by the two modules with poor absolute agreement.

### Left–right eye level of symmetry

**Figure 3:**
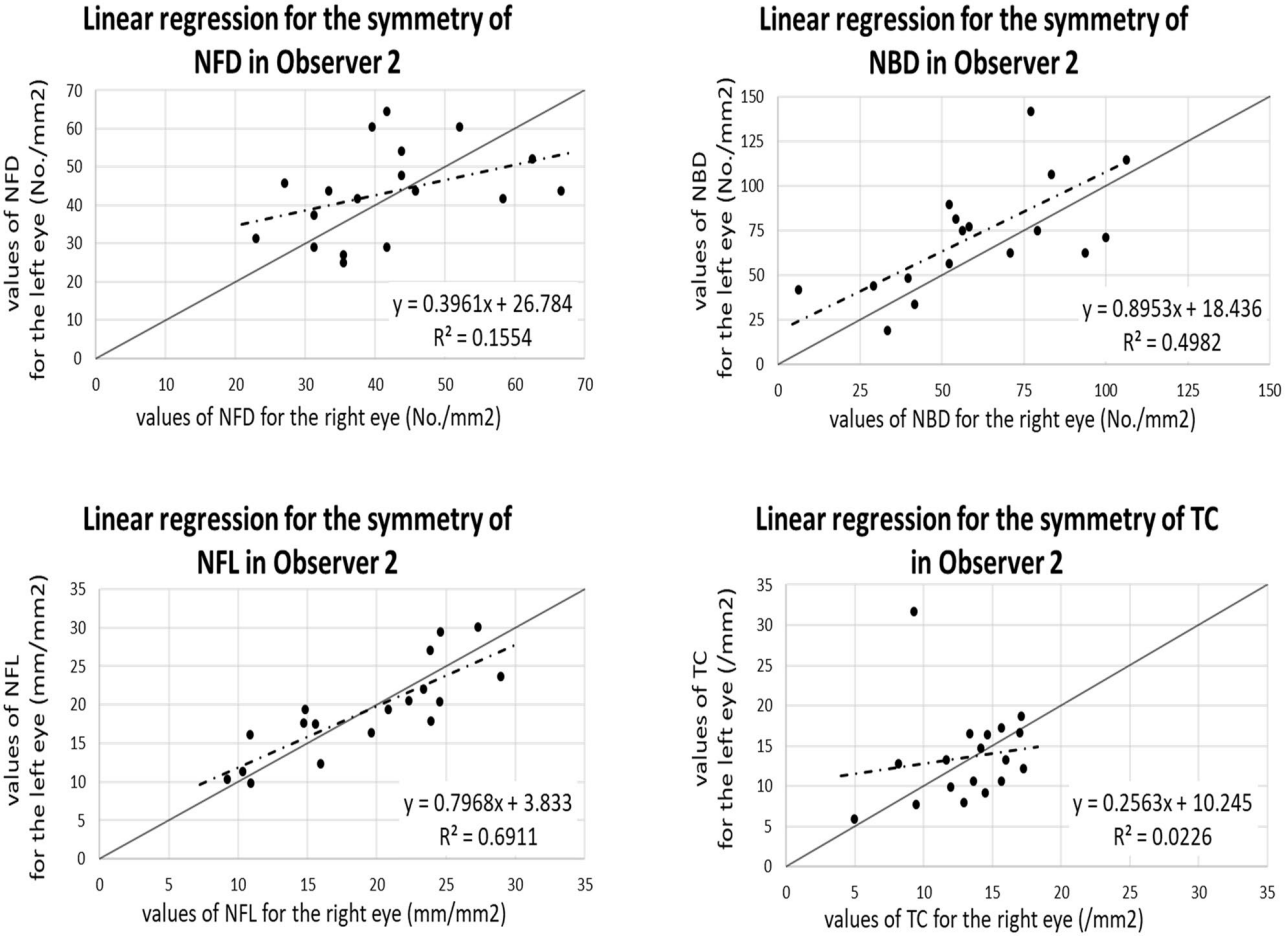
Regression plots of each sub-basal corneal nerve plexus parameters between the right eye and left eye in observer 2 using CCM. Solid in each plot represents the equivalence line while dotted line in each plot represents the Spearman regression line. *NFD* nerve fiber density, *NBD* nerve branch density, *NFL* nerve fiber length, *TC* tortuosity coefficient.

**Figure 4:**
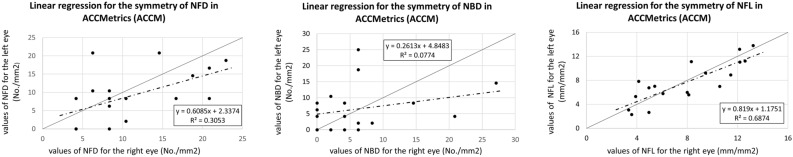
Regression plots of each sub-basal corneal nerve plexus parameters between the right eye and left eye using ACCMetrics (ACCM). Solid in each plot represents the equivalence line while dotted line in each plot represents the Spearman regression line. *NFD* nerve fiber density, *NBD* nerve branch density, *NFL* nerve fiber length.

The left–right eye level of symmetry using CCM and ACCM was also assessed. The values of measurements were summarized in Table 1, and the results of ICC and SpCC calculation were summarized in Table 3. The right and left eyes of each subject were supposed to show similar results when a single module was used for image analysis. Since the CCM measurements from observer 2 in the original group demonstrated the most consistent results among the 3 observers, CCM data derived from observer 2 and ACCM data were used to assess the level of symmetry, and satisfactory results of NFL were found (SpCC = 0.86, ICC = 0.83 for CCM; SpCC = 0.83, ICC = 0.83 for ACCM). Figure 3 depicted the correlation between measurements of the left and right eyes by observer 2, who had the highest intra-observer repeatability in the original group. Figure 4 depicted the correlation between measurements of the two eyes using ACCM.

## Discussion

We examined the performance of beginner observers in quantitative corneal neuroanalysis using manual module and automated module. All observers showed good intra-observer repeatability using CCM when measuring all SCNP parameters, except for TC. Two observers demonstrated especially excellent repeatability, indicating the quality of interpretation may still be observer-dependent. Compared with other parameters, NFL measurement had the best inter-observer reliability and left–right eye level of symmetry based on results by the senior observers. Even when data from both original and additional groups were used, only NFL showed a good inter-observer reliability. The values of all parameters measured by ACCM were significantly lower than that by CCM module, and the results between CCM and ACCM were neither consistent nor comparable.

Based on our results, the intra-observer repeatability of NFD and NBD measured by CCM were satisfactory, and the repeatability of NFL was excellent. As mentioned above, we only provided a brief training of 20 images to the 7 observers before formal evaluation. Our result indicates that, with limited training, SCNP measurements obtained by beginner observers using CCM module seem acceptable. Thus, recruitment of beginner observers could be considered when large-volume corneal image interpretation is needed, and a strict requirement for experience level may not be necessary to pursue a satisfactory result.

Previous literatures did not conclude which SCNP parameter obtained by CCM has the highest repeatability^19–21^. Petropoulos IN et al. demonstrated good repeatability of all major SCNP parameters obtained by CCM except for NBD^19^. However, other studies suggested that only NFL had high inter-observer repeatability across healthy and diseased patients^20,21^. Some studies even stated that corneal nerve evaluation by IVCM should only focus on NFL due to its high reproducibility and validity^20^. In the current study, NFL was the only parameter showing good inter-observer reliability. Furthermore, when evaluating the left–right eye level of symmetry, only NFL showed excellent SpCC values in all observers using both CCM and ACCM. Accordingly, our results suggested that NFL may be the most reliable parameter when evaluating SCNP morphology.

The reason for a better intra- and inter-observer reliability of NFL measurements comparing to NFD and NBD measurements remains elusive. One possible explanation is the unclear operational definition for measuring NFD and NBD^24^, which may lead to subjective interpretation and thus great differences in results obtained by different observers. On the contrary, the measurement of NFL does not involve differentiating the main or branch nerve fiber, and is usually not influenced by the observer’s own judgement. This measurement minimizes the subjective factor in the evaluation process, which might be the reason for its higher repeatability. As for TC, values of TC are calculated based on the observer’s depiction of how tortuous the main fibers are. Therefore, the values of TC reported may easily vary, and it was not surprising that both low intra- and inter-observer repeatability of TC were low.

The currently available tools for corneal neuroanalysis include manual module (CCM), semimanual module (Neuron J), and fully automated module (ACCM). A study has compared NFL values measured using all three modules^22^, and implied that the values of NFL derived from CCM was greater than that from the others. Similarly, our results showed greater values of NFD, NBD, and NFL measured using CCM module (TC was not compared as ACCM could not calculate TC). Our result revealed a huge difference between the results of manual module and automated module, also reported in previous studies^25,26^. The low ICC values further confirmed this observation^27^. In our study, the NFL values obtained using ACCM were not only significant lower compared to results by CCM but also lower than past reports^22,28^. A possible explanation is the higher requirement for image quality when using ACCM to calculate NFL, as defocused nerve on the images cannot be captured by automated software. In contrast, for human observer, delineation of the nerve was relatively unaffected by a blurry background. Although we included images after quality selection, the selected images might not have been optimal for automatic analysis. In addition, the results by ACCM can be easily affected by the optical quality of the images (brightness, contrast, sharpness, etc.) (Supplementary Fig. S1), making the reliability of this method more settings-dependent.

Recently, machine learning algorithms showed excellent performance in medical image analysis. Several artificial intelligence-based methods have been developed to improve the less-than-ideal results by ACCM^29,30^. In two prior studies, NFL measured using machine learning techniques was comparable to that obtained using manual method and was significantly better than results by ACCM^29,30^. Therefore, in addition to outsourcing this task to beginner observers, the utilization of artificial intelligence-based automated methods may be another option to reduce the labor- and time-associated cost of training professional image-readers.

There were several limitations of the current study. First, as our subjects were all healthy young males, the results may not be applicable to diseased eyes^3,31–33^. Second, corneal characteristics may differ based on ethnicity, so our results may not be generalizable for Western populations. For instance, Asians have smaller anterior segments and higher prevalence of myopia^34–36^, both of which may affect the SCNP parameters^37^. The reported values of normal SCNP measurements also varied across different ethnicities in past reports^24,38,39^. Similarly, our results cannot serve as the reference for normative values of SCNP measurements. Third, the cohort size was relatively small in our study, and they were all healthy young males. Therefore, the generalizability of our results to healthy populations with different demographic characteristics or diseased populations remains unknown. Lastly, our study did not include performance of quantitative neuroanalysis by an experienced expert. Thus, the validity of the beginner observers’ results cannot be confirmed due to the lack of ground truth provided by an expert observer. However, evaluation of the validity of beginner observers may be our next goal in establishing a reliable and accurate method for large-volume neuroanalysis.

In conclusion, without extensive training, beginners may still achieve acceptable performance in manual quantitative corneal neuroanalysis, and NFL had the best reliability among all SCNP parameters. Automated module, although convenient, cannot yet replace manual work as it may lead to underestimation of the measurements. Before a more accurate and reliable automated method is established, human resource may still be the main force for neuroanalysis, and our results may serve as the basis for future work and studies on large-volume image interpretation for corneal neuropathies.

## Materials and methods

### Study subjects

The study was approved by National Taiwan University Hospital Ethics Committee and was conducted in accordance with the Declaration of Helsinki. Healthy male volunteers without corneal diseases, peripheral neuropathy or diabetes were recruited for IVCM imaging. Those who had history of contact lens use or refractive surgeries were excluded. Before enrollment, both eyes of each subject were examined by slit-lamp biomicroscopy and were confirmed to be clinically healthy. Written informed consent was obtained from all patients.

### In vivo corneal confocal microscopy

IVCM scan (Heidelberg Retinal Tomograph III (HRT III), Heidelberg Engineering GmbH, Heidelberg, Germany) was performed on all subjects. This IVCM uses a 670-nm wavelength helium–neon diode laser which was proven to be safe for ocular usage. An X63 objective lens with a numerical 0.9 um working intervals relative to the anterior surface of applanating cap (TomoCap; Heidelberg Engineering GmbH) was used. The obtained size of the 2-dimentional image products was 384 × 384 um, with a transverse optical resolution of 10 um per pixel.

The examinations were performed by an experienced technician following published protocols^11,40–42^. Briefly, topical anesthetic was applied to the corneal surface, and a viscous gel medium was then applied to the corneal surfaces 5 min later, which permitted a visual gel bridge between the sterile cap on the microscope objective lens and the surface of the central cornea. To ensure examination of the central cornea, the subjects were instructed to fixate on the flashing light of the instrument. We used the interface between the corneal epithelium and Bowman’s layer as a reference point, such that the examiner could easily find the image with the highest contrast on the SCNP.

In this study, we used a “volume scan mode” to capture a set of 40 automatically obtained images at each examination^21^. Around 6 to 8 examinations were repeated for each eye, and both eyes of each subject were examined. The overall examination took about 3–5 min. Three images were selected from each eye of each subject, and the selection was based on the depth, focus position, and contrast of the images. Details of the image selection criteria was summarized in prior study^43^. Images with even distribution of SCNP on the whole area were also the selection criteria.

### Image analysis for SCNP parameters

Seven beginner observers with similar research background were recruited to analyze IVCM images and were divided into two groups. The original group consisted of 3 observers, who performed the data analysis from December 2019 to March 2020, and the additional group consisted of 4 observers, who performed the data analysis from June 2021 to July 2021. All beginner observers had no previous experience with corneal neuroanalysis. Both manual module (CCMetrics, CCM, Manual tracing of nerve fibers, Manchester University, Manchester, UK) and fully automated module (ACCMetrics, ACCM, Automated tracing of nerve fibers, Manchester University, Manchester, UK)^44^ were used. Before the study started, all observers were instructed to practice corneal nerve quantification using CCM on 20 images obtained from IVCM that were not used in formal evaluation.

**Figure 5:**
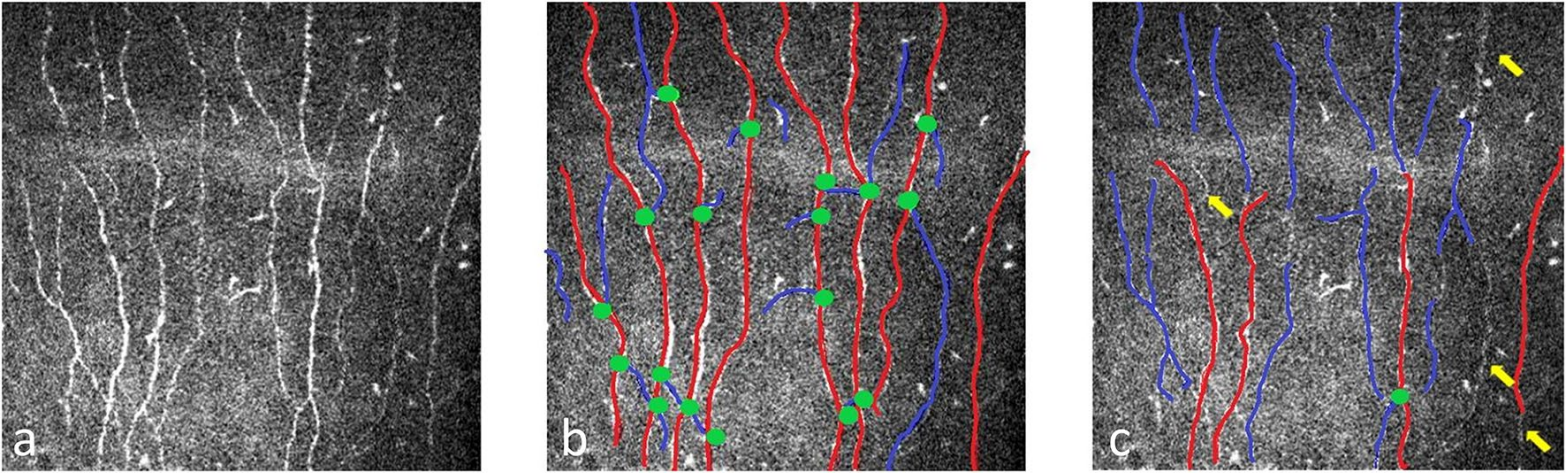
Illustrations representative of **(a)**, original image from Heidelberg Retinal Tomograph III (HRT III) showing the distribution of sub-basal corneal nerve plexus. **(b)**, an analyzed image using manual software from CCMetrics (CCM). **(c)**, an analyzed image using fully automated software ACCMetrics (ACCM). In both **(b,c)** images, red lines represented main fiber, which was used to calculate nerve fiber density (NFD); green dots represented the junction between the main fiber and the branch fiber (the blue line), which was used to calculate nerve branch density (NBD); NFL, nerve fiber length, represented the total length of red lines and blue lines in the image. The data from CCM **(b)** had obviously higher NFD, NBD and higher NFL compared to data from ACCM **(c)**. Yellow arrows in **(c)** indicated the missing nerve fibers calculated by ACCM.

The quantitative SCNP parameters measured in the study were: (1) corneal nerve fiber density (NFD) (numbers per square millimeter), (2) corneal nerve branch density (NBD) (numbers per square millimeter), (3) corneal nerve fiber length (NFL) (millimeters per square millimeter), and (4) corneal tortuosity coefficient (TC) (Fig. 5)^19,21,39^. NFD is the total number of main nerve fibers (NF) per frame divided by the surface area of the frame in square millimeters (area = 0.16 mm^2^; Fig. 5). NBD is the total number of main nerve branches (NBs, defined as the nerve branches that stem from an NF) divided by the surface area of the image frame. NFL is the total length of NFs, NBs, and secondary NBs (branches that stem from an NB) per frame. TC is a mathematical computation of the tortuosity of NF previously described by Kallinikos et al.^3^, which is independent of the angle of the nerve in the image. A straight nerve has the value of 0 in TC, and the value of TC increases when tortuosity of the NF increases. NFD, NBD, and NFL were measured using both modules, while TC was only measured by CCM module as ACCM did not provided analysis of TC.

There were two sessions of evaluation. In the first evaluation, each observer used CCM and ACCM to evaluate the randomly distributed IVCM images of the subjects. The second evaluation was performed 14 days after completion of the first evaluation, and the observers were asked to repeat analysis for all masked images after further randomization. Only CCM was used in the second evaluation, as the ACCM module was fully automated and the result would not change.

The intra-observer repeatability of individual observer was assessed using CCM data from both sessions of evaluations, and all 7 observers were examined for their individual repeatability. The inter-observer reliability, left–right eye level of symmetry, and inter-module agreement were examined using data derived from the first evaluation by the 3 observers in the original group. The inter-observer reliability was assessed using CCM measurements between any two observers, and the level of symmetry was the correlation between measurements derived from the right and the left eye of the same subject.

### Statistical analysis

Statistical analysis was performed using Microsoft Office Excel 2019 (Microsoft, WA, USA) and SPSS version 25 (IBM, Chicago, IL, USA). Numerical data were presented as mean ± SD. Differences between measurements using CCM module and ACCM module were evaluated by paired *t* tests. The intra-observer, inter-observer repeatability and the level of left–right eye symmetry were presented as the Spearman’s rank-sum correlation coefficient (SpCC) for correlation and intra-class correlation coefficient (ICC) for absolute agreement. ICC class (2,1) was used because it was a more conservative estimate of reliability with less bias^27,45^. SpCCs and ICCs were considered excellent if the values were between 0.80 and 1.00 and good if the values were between 0.60 and 0.79. Bland–Altman plots were generated to facilitate appreciation of the extent of intra-observer discrepancy of each morphological parameter^46,47^. Linear regression plots were generated for the symmetry between two eyes. Bland–Altman plot and liner regression plot were used to show the absolute agreement and correlation between CCM module and ACC module, and the observer in the original group who had the highest intra-observer repeatability in CCM module was chosen for comparison.

## Supplementary Information

Supplementary Legends.

Supplementary Table S1.

Supplementary Table S2.

Supplementary Figure S1.

## References

[1] Guthoff RF, Wienss H, Hahnel C, Wree A (2005). Epithelial innervation of human cornea: A three-dimensional study using confocal laser scanning fluorescence microscopy. Cornea.

[2] Edwards K (2012). Utility of corneal confocal microscopy for assessing mild diabetic neuropathy: Baseline findings of the LANDMark study. Clin. Exp. Optom..

[3] Kallinikos P (2004). Corneal nerve tortuosity in diabetic patients with neuropathy. Investig. Ophthalmol. Vis. Sci..

[4] Villani E, Galimberti D, Viola F, Mapelli C, Ratiglia R (2007). The cornea in Sjogren's syndrome: An in vivo confocal study. Investig. Ophthalmol. Vis. Sci..

[5] Benítez-Del-Castillo JM (2007). Relation between corneal innervation with confocal microscopy and corneal sensitivity with noncontact esthesiometry in patients with dry eye. Investig. Ophthalmol. Vis. Sci..

[6] Mead OG, Tighe S, Tseng SCG (2020). Amniotic membrane transplantation for managing dry eye and neurotrophic keratitis. Taiwan J. Ophthalmol..

[7] Linna TU (2000). Effect of myopic LASIK on corneal sensitivity and morphology of subbasal nerves. Investig. Ophthalmol. Vis. Sci..

[8] Avunduk AM, Senft CJ, Emerah S, Varnell ED, Kaufman HE (2004). Corneal healing after uncomplicated LASIK and its relationship to refractive changes: A six-month prospective confocal study. Investig. Ophthalmol. Vis. Sci..

[9] Tai YC, Sun CC (2019). Effects of flap diameter on dry eye parameters and corneal sensation after femtosecond laser-assisted LASIK. Taiwan J. Ophthalmol..

[10] Fernyhough P, Calcutt NA (2010). Abnormal calcium homeostasis in peripheral neuropathies. Cell Calcium.

[11] Mehra S (2007). Corneal confocal microscopy detects early nerve regeneration after pancreas transplantation in patients with type 1 diabetes. Diabetes Care.

[12] Tavakoli M (2009). Corneal confocal microscopy: A novel noninvasive means to diagnose neuropathy in patients with Fabry disease. Muscle Nerve.

[13] Tavakoli M (2012). Corneal confocal microscopy detects small-fiber neuropathy in Charcot-Marie-Tooth disease type 1A patients. Muscle Nerve.

[14] Chen WL (2009). In vivo confocal microscopic findings of corneal wound healing after corneal epithelial debridement in diabetic vitrectomy. Ophthalmology.

[15] Chu H-S, Huang S-L, Chen W-L (2020). In-depth thinking about the diagnostic methods and treatment strategies for the corneal nerves in ocular surface disorders. Curr. Ophthalmol. Rep..

[16] Grupcheva CN, Wong T, Riley AF, McGhee CN (2002). Assessing the sub-basal nerve plexus of the living healthy human cornea by in vivo confocal microscopy. Clin. Exp. Ophthalmol..

[17] Ranno S, Fogagnolo P, Rossetti L, Orzalesi N, Nucci P (2011). Changes in corneal parameters at confocal microscopy in treated glaucoma patients. Clin. Ophthalmol. (Auckland).

[18] Chin JY (2020). Validation of the use of automated and manual quantitative analysis of corneal nerve plexus following refractive surgery. Diagnostics..

[19] Petropoulos IN (2013). Repeatability of in vivo corneal confocal microscopy to quantify corneal nerve morphology. Cornea.

[20] Efron N (2010). Repeatability of measuring corneal subbasal nerve fiber length in individuals with type 2 diabetes. Eye Contact Lens Sci. Clin. Pract..

[21] Hertz P (2011). Reproducibility of in vivo corneal confocal microscopy as a novel screening test for early diabetic sensorimotor polyneuropathy. Diabet. Med..

[22] Dehghani C (2014). Fully automated, semiautomated, and manual morphometric analysis of corneal subbasal nerve plexus in individuals with and without diabetes. Cornea.

[23] Brady CJ (2014). Rapid grading of fundus photographs for diabetic retinopathy using crowdsourcing. J. Med. Internet Res..

[24] Patel DV, McGhee CN (2009). In vivo confocal microscopy of human corneal nerves in health, in ocular and systemic disease, and following corneal surgery: A review. Br. J. Ophthalmol..

[25] Scarpa F, Grisan E, Ruggeri A (2008). Automatic recognition of corneal nerve structures in images from confocal microscopy. Investig. Ophthalmol. Vis. Sci..

[26] Ferreira A, Morgado AM, Silva JS (2012). A method for corneal nerves automatic segmentation and morphometric analysis. Comput Methods Progr. Biomed..

[27] Shrout PE, Fleiss JL (1979). Intraclass correlations: Uses in assessing rater reliability. Psychol. Bull..

[28] Chen X (2017). An automatic tool for quantification of nerve fibers in corneal confocal microscopy images. IEEE Trans. Biomed. Eng..

[29] Salahouddin T (2021). Artificial intelligence-based classification of diabetic peripheral neuropathy from corneal confocal microscopy images. Diabetes Care.

[30] Williams BM (2020). An artificial intelligence-based deep learning algorithm for the diagnosis of diabetic neuropathy using corneal confocal microscopy: A development and validation study. Diabetologia.

[31] Ahmed A (2012). Detection of diabetic sensorimotor polyneuropathy by corneal confocal microscopy in type 1 diabetes: A concurrent validity study. Diabetes Care.

[32] Darwish T, Brahma A, O'Donnell C, Efron N (2007). Subbasal nerve fiber regeneration after LASIK and LASEK assessed by noncontact esthesiometry and in vivo confocal microscopy: Prospective study. J. Cataract Refract. Surg..

[33] Thia ZZ, Tong L (2019). Update on the role of impression cytology in ocular surface disease. Taiwan J. Ophthalmol..

[34] Qin B (2012). Anterior segment dimensions in Asian and Caucasian eyes measured by optical coherence tomography. Ophthalmic Surg. Lasers Imaging.

[35] Rudnicka AR, Owen CG, Nightingale CM, Cook DG, Whincup PH (2010). Ethnic differences in the prevalence of myopia and ocular biometry in 10- and 11-year-old children: The Child Heart and Health Study in England (CHASE). Investig. Ophthalmol. Vis. Sci..

[36] Chuang AY (2017). How to effectively manage myopia. Taiwan J. Ophthalmol..

[37] Harrison WW (2017). The corneal nerve density in the sub-basal plexus decreases with increasing myopia: A pilot study. Ophthalmic Physiol. Opt..

[38] Poon LY (2018). Effects of age, race, and ethnicity on the optic nerve and peripapillary region using spectral-domain OCT 3D volume scans. Transl. Vis. Sci. Technol..

[39] Tavakoli M (2015). Normative values for corneal nerve morphology assessed using corneal confocal microscopy: A multinational normative data set. Diabetes Care.

[40] Hung K-C (2020). Use of white light in vivo confocal microscopy for the detection of spatial changes in the corneal nerves in cases of early-stage *Acanthamoeba keratitis* with radial keratoneuritis. Indian J. Ophthalmol..

[41] Huang CT (2021). The effect of human platelet lysate on corneal nerve regeneration. Br. J. Ophthalmol..

[42] Huang CJ (2017). Comparison of corneal epitheliotrophic capacities among human platelet lysates and other blood derivatives. PLoS ONE.

[43] Kalteniece A (2017). Corneal confocal microscopy is a rapid reproducible ophthalmic technique for quantifying corneal nerve abnormalities. PLoS ONE.

[44] Dabbah MA, Graham J, Petropoulos I, Tavakoli M, Malik RA (2010). Dual-model automatic detection of nerve-fibres in corneal confocal microscopy images. Med. Image Comput. Comput. Assist. Interv..

[45] Koo TK, Li MY (2016). A guideline of selecting and reporting intraclass correlation coefficients for reliability research. J. Chiropr. Med..

[46] Giavarina D (2015). Understanding Bland Altman analysis. Biochem. Med. (Zagreb.).

[47] Bland JM, Altman DG (1986). Statistical methods for assessing agreement between two methods of clinical measurement. Lancet.

