# Supplementary material for: Comparing the results of manual and automated quantitative corneal neuroanalysing modules for beginners
Source: Sci Rep. 2021 Sep 14;11:18208. doi: 10.1038/s41598-021-97567-y (PMC8440557; doi:10.1038/s41598-021-97567-y)
Supplement: Supplementary file 1 — Supplementary Legends. [file 41598_2021_97567_MOESM1_ESM.docx]

**Supplement legends:**

**Supplementary Table 1:** The CCMetrics (CCM) values of NFD, NBD and NFL in the first and the second evaluations from the additional group.

**Supplementary Table 2:** The Spearman correlation coefficient (SpCC) and intraclass correlation coefficient (ICC) to inter-module agreement between CCMetrics and ACCMetrics for the additional group.

**Supplementary Figure 1:** Examples of analyzed images from automated module, ACCMetrics (ACCM) influenced by the image settings. (a), an original image. (b), an original analyzed image by manual module, CCMetrics. (c), an original analyzed image by ACCM. (d)~(i), adjusted images with different optical qualities (brightness, contrast, sharpness, etc) analyzed by ACCM. Results from ACCM were highly influenced by the image settings. In all images, red lines represented main fiber to calculate nerve fiber density (NFD); green dots represented the junction between the main fiber and the branch fiber (the blue line) to calculate nerve branch density (NBD); Nerve fiber length (NFL) represented the total length of red lines and blue lines in the image.
