# Supplementary material for: Comparing the results of manual and automated quantitative corneal neuroanalysing modules for beginners
Source: Sci Rep. 2021 Sep 14;11:18208. doi: 10.1038/s41598-021-97567-y (PMC8440557; doi:10.1038/s41598-021-97567-y)
Supplement: Supplementary file 2 — Supplementary Table S1. [file 41598_2021_97567_MOESM2_ESM.docx]

**Supplementary Table 1:** The CCMetrics (CCM) values of NFD, NBD and NFL in the first and the second evaluations from the additional group

| **Manual Method** |  | **NFD (No./mm2)** | **NBD (No./mm2)** | **NFL (mm/mm2)** | **TC** |
| --- | --- | --- | --- | --- | --- |
| CCMetrics, Observer 4 | Evaluation 1 | 19.21±6.36 | 41.72±19.02 | 15.61±5.01 | 14.17±4.80 |
|  | Evaluation 2 | 18.49±6.55 | 33.07±18.64 | 13.99±5.09 | 12.72±4.20 |
| CCMetrics, Observer 5 | Evaluation 1 | 18.11±6.66 | 24.88±17.24 | 14.84±5.96 | 13.05±4.96 |
|  | Evaluation 2 | 17.65±6.78 | 24.36±17.36 | 14.47±5.83 | 12.94±4.56 |
| CCMetrics, Observer 6 | Evaluation 1 | 22.22±6.66 | 35.01±19.34 | 15.52±5.67 | 13.31±4.20 |
|  | Evaluation 2 | 25.00±7.32 | 40.62±20.97 | 15.67±5.52 | 12.70±4.14 |
| CCMetrics, Observer 7 | Evaluation 1 | 17.42±8.26 | 79.74±46.68 | 20.15±6.88 | 11.23±4.62 |
|  | Evaluation 2 | 17.07±8.13 | 91.38±49.66 | 21.23±6.65 | 11.55±4.46 |
| Results are expressed as Mean ± SD | | | | | |
| NFD (nerve fiber density) is measured in number of fibers/mm2. NBD (nerve branch density) is measured in number of branch points on the main fibers/mm2. NFL (nerve fiber length) is measured in total length of fiber (mm/mm2). TC (tortuosity coefficient) is measured in main fiber average tortuosity. | | | | | |
