# Supplementary material for: Comparing the results of manual and automated quantitative corneal neuroanalysing modules for beginners
Source: Sci Rep. 2021 Sep 14;11:18208. doi: 10.1038/s41598-021-97567-y (PMC8440557; doi:10.1038/s41598-021-97567-y)
Supplement: Supplementary file 3 — Supplementary Table S2. [file 41598_2021_97567_MOESM3_ESM.docx]

**Supplementary Table 2:** The Spearman correlation coefficient (SpCC) and intraclass correlation coefficient (ICC) to inter-module agreement between CCMetrics and ACCMetrics for the additional group.

| **Inter-module agreement for the additional group** | | | | | | |
| --- | --- | --- | --- | --- | --- | --- |
|  | **NFD** |  | **NBD** |  | **NFL** |  |
|  | **SpCC** | **ICC** | **SpCC** | **ICC** | **SpCC** | **ICC** |
| ACCM vs Observer 4 | 0.57 | 0.39 | 0.50 | 0.15 | 0.88* | 0.36 |
| ACCM vs Observer 5 | 0.50 | 0.40 | 0.58 | 0.30 | 0.90* | 0.43 |
| ACCM vs Observer 6 | 0.50 | 0.28 | 0.60 | 0.19 | 0.90* | 0.39 |
| ACCM vs Observer 7 | 0.75 | 0.56 | 0.55 | 0.07 | 0.78 | 0.21 |
| * represents an excellent correlation when values >0.8.  Abbreviation: NFD, nerve fiber density. NBD, nerve branch density. NFL, nerve fiber length. TC, tortuosity coefficient. SpCC, Spearman correlation coefficient. ICC, intraclass correlation coefficient. ACCM, ACCMetrics module. | | | | | | |
