# Supplementary figures and images for: Comparing the results of manual and automated quantitative corneal neuroanalysing modules for beginners
Source: Sci Rep. 2021 Sep 14;11:18208. doi: 10.1038/s41598-021-97567-y (PMC8440557; doi:10.1038/s41598-021-97567-y)

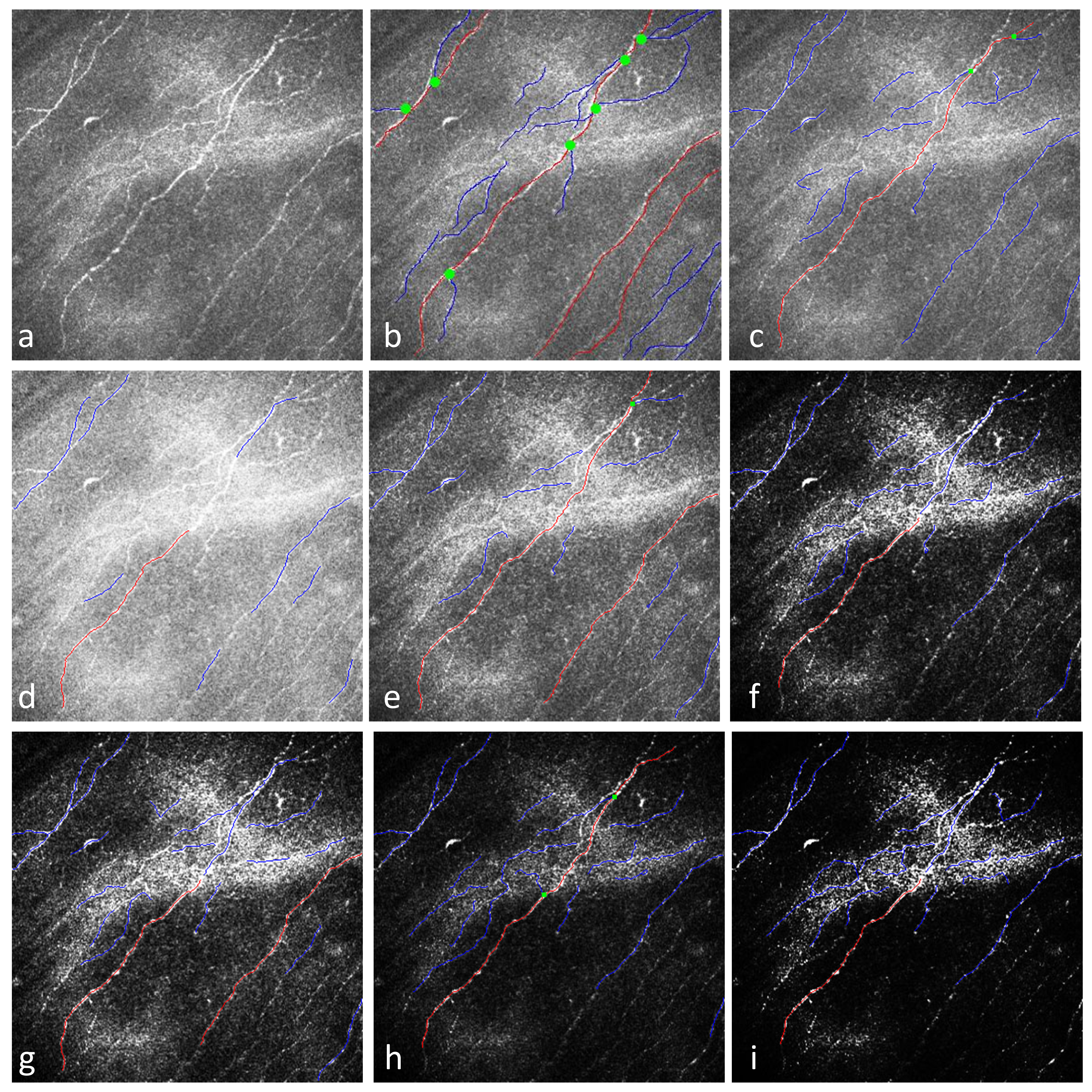

Supplement: Supplementary file 4 — Supplementary Figure S1. [file 41598_2021_97567_MOESM4_ESM.tif]
